# Smartphone-Based Auditory Motion Stimulation for Hemispatial Neglect: Development and Usability Study 

**DOI:** 10.2196/82442

**Published:** 2025-12-01

**Authors:** Tobias Nef, Nora Geiser, Brigitte C Kaufmann, Noora Emilia Angelva, Nic Krummenacher, Michael Single, Dario Cazzoli, Thomas Nyffeler

**Affiliations:** 1 Gerontechnology and Rehabilitation Group ARTORG Center for Biomedical Engineering Research University of Bern Bern Switzerland; 2 Department of Neurology Bern University Hospital Bern Switzerland; 3 Graduate School for Health Sciences University of Bern Bern Switzerland; 4 Neurocenter University of Lucerne, University Teaching and Research Hospital, Lucerne Cantonal Hospital Lucerne Switzerland; 5 Faculty of Behavioral Sciences and Psychology University of Lucerne Bern Switzerland; 6 Faculty of Health Sciences and Medicine University of Lucerne Lucerne Switzerland

**Keywords:** hemispatial neglect, auditory motion stimulation, spatial audio, head-related transfer function, mobile health, neurorehabilitation, usability

## Abstract

**Background:**

Hemispatial neglect affects approximately 33% of patients after acute stroke and is linked to poor recovery. Auditory motion stimulation, particularly using spatial cues, can enhance spatial awareness and has shown promise in experimental settings.

**Objective:**

This study aimed to develop and evaluate Neglect Radio, a smartphone app that delivers auditory motion stimulation, focusing on sound realism and usability in healthy volunteers.

**Methods:**

Two auditory motion rendering methods, dynamic stereo volume control and standardized head-related transfer functions (HRTFs), were implemented in a mobile app streaming public radio content. In an online study, 37 healthy volunteers rated spatial realism (0-100 scale) of 5 audio sources in 3 conditions: static stereo, volume control motion, and HRTF-based motion. Independent samples 1-tailed *t* tests compared realism scores. Ten participants tested the app for 15 minutes and completed the System Usability Scale (SUS).

**Results:**

HRTF-based audio was rated as being significantly more realistic than volume control (t_72_=3.722; *P*<.001), and both motion conditions scored significantly higher than static stereo. The mean SUS score was 86.5 (SD 6.9), exceeding the 68-point threshold for above-average usability and a rating of “excellent.”

**Conclusions:**

Neglect Radio successfully produced realistic spatial auditory motion and achieved excellent usability ratings in healthy participants. This smartphone-based platform is scalable, accessible, and engaging, with potential to complement hemispatial neglect rehabilitation. However, clinical efficacy in stroke patients with hemispatial neglect has not yet been evaluated and requires confirmation in controlled trials.

## Introduction

Hemispatial neglect after stroke remains a major challenge in neurorehabilitation [1]. Patients with this attentional disorder fail to attend and respond to stimuli in their contralesional (often left) visual field despite having intact primary visual processing [2]. Notably, hemispatial neglect is very common after stroke, affecting roughly one-third of patients after acute stroke [3]. Traditional rehabilitation methods focus on visual or sensorimotor interventions, yet recent research has increasingly explored multisensory stimulation, particularly auditory cues paired with visual stimuli, as a potential mechanism to compensate for visual deficits [4]. Auditory stimulation has been shown to influence spatial perception and attention, potentially activating compensatory neural pathways [5,6].

In one of the foundational studies in this area, Bolognini et al [7] examined the sound-induced flash illusion in patients with hemianopia and spatial neglect. Their findings indicated that auditory stimuli could enhance visual perception, reinforcing the role of multisensory integration in rehabilitation. Building on this, Zigiotto et al [8] demonstrated that multisensory stimulation protocols improved spatial awareness in patients with neglect. This highlights the concept that auditory signals can serve as external “anchors,” enhancing awareness of the neglected visual space. An early study by Lewald et al [9] provided direct evidence that passive auditory stimulation can enhance visual perception in patients. By exposing individuals to simple sound cues, the authors demonstrated significant improvements in spatial attention. Furthermore, music-based therapy using nonspatial auditory stimuli has shown positive effects on neglect [10-12], suggesting that even passive music can engage attention. Building on these insights, auditory motion stimulation can be viewed as music therapy enhanced with spatial cues.

Kaufmann et al [13] conducted a proof-of-concept study showing that auditory motion stimulation reduces neglect symptoms after right-hemispheric stroke. They observed a short-term improvement in neglect severity after just a single session of auditory motion stimulation. Following this line of research, Geiser et al [14] conducted a controlled study with 30 patients, reporting that a 3-week auditory motion stimulation training program was feasible, well tolerated, and led to significant improvements in neglect symptoms by discharge. In auditory motion stimulation, patients listen to music through headphones, with the music moving repeatedly at a constant speed from the ipsilesional toward the contralesional hemispace (or vice versa for right-sided neglect) [13,15]. While the clinical efficacy of auditory cueing has been demonstrated in prior studies [14], this work focuses on the technical development and preliminary evaluation of a smartphone-based intervention.

This emerging evidence forms the motivation for a novel rehabilitation approach that we call Neglect Radio—a targeted auditory motion system designed to enhance spatial awareness and mitigate symptoms of hemispatial neglect. Based on the pilot studies by Kaufmann et al [13] and Geiser et al [15], as well as the controlled-group study by Geiser et al [14], the objective of this work was to develop a smartphone app that delivers auditory motion stimulation directly to patients. The 15-to-45-minute range was chosen to balance effective training time with patient endurance, reflecting typical therapy session lengths. Accordingly, this manuscript focuses on three key aspects: (1) generating the perceptual illusion of a moving sound source; (2) selecting appropriate and engaging audio content; and (3) implementing the Neglect Radio app for smartphone platforms.

## Methods

This study follows the CONSORT-EHEALTH reporting guidelines (V.1.6.1) for trials of eHealth and mHealth interventions, adapted here for a preliminary development and usability study of a mobile app.

### Creating the Illusion of a Moving Sound Source

In the Neglect Radio smartphone app, it is essential to generate a convincing auditory illusion of a sound source moving from right to left in patients with left-sided neglect (or left to right in patients with right-sided neglect). This effect can be achieved using two primary approaches, dynamic volume control of stereo channels and simulation of a virtual sound source using head-related transfer functions (HRTFs).

#### Dynamic Volume Control of Stereo Channels

One simple way to simulate movement is to adjust the volume of the right and left stereo channels. By gradually decreasing the volume on one side while increasing it on the other, the perception of motion can be effectively induced. This approach relies on principles of binaural hearing, where the human auditory system interprets volume differences between both ears as an indication of sound source direction. Relevant studies on binaural hearing, such as the one by Blauert [16], emphasize the importance of interaural level differences in spatial hearing. Dynamic volume control is most effective for higher-frequency sounds, where the head acts as an acoustic shadow. Previous psychoacoustic studies have demonstrated that smooth transitions in volume control significantly enhance the illusion of motion [17].

#### Simulation of a Virtual Sound Source Using HRTFs

HRTFs describe how sound waves interact with the outer ear, head, and shoulders before reaching the eardrum. Filtering audio signals through HRTFs creates a realistic perception of spatial movement. Wightman and Kistler [18] showed that HRTFs externalize virtual sound sources, making them appear to come from specific spatial locations. Begault [19] further demonstrated that individualized HRTFs improve localization accuracy and immersion. Although real-time HRTF rendering is computationally demanding, recent advances in digital signal processing now allow efficient spatial audio generation.

Both methods have advantages and limitations. The volume control method is computationally simple and widely supported by standard stereo playback systems, but it lacks spatial depth. HRTF simulation, on the other hand, provides a more immersive experience, but requires individualized filtering and additional processing power. The choice of method depends on the application context. For scenarios in which computational resources are limited, the volume control method offers a practical solution. In contrast, applications demanding high spatial realism, such as virtual reality or advanced auditory displays, benefit from HRTF-based rendering. Headphones are essential in both cases, as they deliver separate signals to each ear. For scalable use in neglect therapy, individualized HRTFs are impractical. This raises the practical question of whether standardized HRTFs provide sufficient perceptual benefit compared to volume control.

### Spatial Realism Experiment

A total of 37 healthy adult volunteers were recruited through university mailing lists at the University of Bern (Switzerland). Inclusion criteria required normal hearing and fluency in German or English. Participation was anonymous and voluntary, and no compensation was provided.

The study required the use of stereo headphones. Before beginning, participants were presented with a mandatory checkbox confirming that they were wearing headphones; only those who confirmed were able to proceed. Participants were instructed to adjust their device volume to a comfortable, medium level before starting. Because the study was conducted online, absolute sound levels could not be standardized, but the relative levels across the 3 auditory conditions were kept identical in all stimuli.

Each participant listened to audio samples of 5 everyday sound sources (a car, a crying baby, footsteps, a singing child, and a dog barking). Each sound was presented in three formats: (1) static stereo (no motion), (2) dynamic stereo volume control simulating left-to-right movement at 10° per second, and (3) HRTF-based audio simulating movement at 10° per second. Stimuli were presented in randomized order, and participants were not informed of the rendering method. In total, each participant rated 15 files. Realism was rated on a 0 to 100 scale (0=completely unrealistic, 100=highly realistic) using an online questionnaire interface.

Because all participants rated all 3 auditory conditions, a repeated-measures ANOVA was conducted to compare realism ratings across conditions. The Mauchly test indicated a violation of sphericity; therefore, Greenhouse-Geisser corrections were applied. Post hoc pairwise comparisons with Bonferroni adjustment were conducted to test differences between HRTF, dynamic volume control, and static stereo.

The sample of 37 participants was chosen to align with a previous perceptual study in spatial audio research that included 20 to 40 participants and was sufficiently powered to detect medium-to-large within-subject effects [20]. The subsequent usability evaluation was conducted with 10 participants, following established usability engineering guidelines suggesting that 5 to 10 users are sufficient to identify the majority of usability issues [21-23].

### Selecting Engaging Audio Content

To maximize patient motivation and adherence, the selection of the audio content is a relevant factor. Two possible types of content for the audio signal were considered. The first type was realistic soundscapes, including everyday auditory experiences such as footsteps moving from right to left or a car driving past. Such sounds align with real-world expectations, making them highly effective in evoking spatial perception. Studies in ecological acoustics [24] suggest that familiarity with natural sounds enhances spatial awareness and immersion. The second type was entertaining content, including music, entertainment programs, and informational broadcasts and recordings. While not inherently directional, these sounds can be spatialized to create the illusion of a sound source travelling from the right to the left side. Research on immersive audio [25] indicates that even abstract audio elements, when processed through spatial techniques, can evoke a sense of movement.

Both approaches offer valuable contributions to the Neglect Radio concept. Realistic sounds provide strong spatial cues grounded in everyday experiences, while entertaining content potentially enhances engagement and listener retention. Based on the report by Kaufmann et al [13], we assume that repeatedly listening to realistic soundscapes alone, despite their significant spatial effect, would become somewhat monotonous over extended periods. Therefore, we suspect that effective everyday audio stimulation should contain entertaining content such as radio programs.

Listening habits among older adults support this choice. Surveys show that more than 75% of adults older than 30 years listen to radio weekly, and those over 60 often listen for several hours daily [26,27]. Many individuals older than 65 years continue to cherish personal music collections, often consisting of CDs or digital playlists featuring music that reflects the soundtrack of their youth. Research by Hays and Minichiello [28] suggests that listening to familiar music enhances cognitive function and emotional well-being in older adults. For practical and legal reasons, our app focuses on streaming publicly available web radio programs rather than personal music files, ensuring a wide variety of content without copyright barriers.

### Implementing the Neglect Radio Smartphone App

Based on the considerations described in the Creating the Illusion of a Moving Sound Source and Selecting Engaging Audio Content sections, as well as previous experiments [13-15], the Neglect Radio smartphone app was designed to fulfill the following functional requirements:

1. Streaming of web radio stations
2. Adjustable movement speed of the sound source (5-40°/s; default 10°/s; this range was chosen based on pilot testing to ensure the motion is perceivable yet comfortable for listeners)
3. Integration of spatial audio via HRTFs
4. Multilingual user interface and content (initially German, French, and English)
5. Region-based station selection to ensure culturally relevant material
6. Availability on both iOS and Android devices

**Figure 1:**
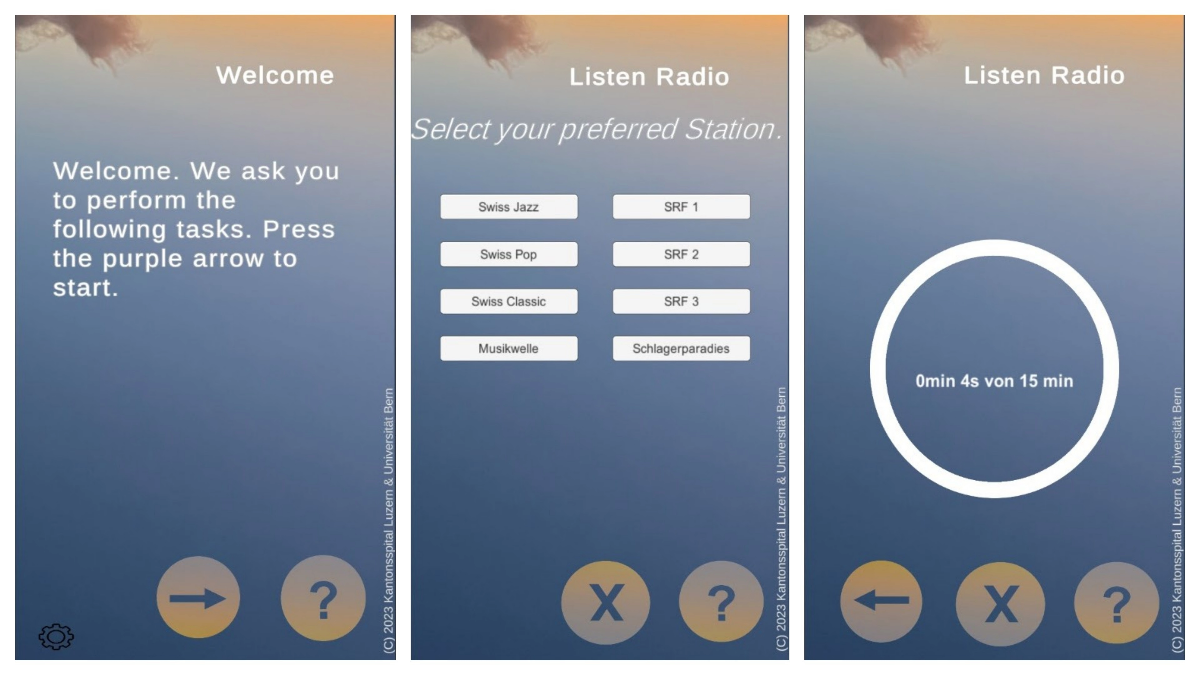
Screenshots of the smartphone app. Participants can select the language (German, English, or French). They can then select a radio station from the laut.fm screening service and listen to the spatial presentation of the audio. While listening, the total listening time in minutes is shown on screen.

Based on functional requirement 6, we decided to use the Unity3D (version 5.0; Unity Technologies) programming environment for app development, allowing us to deploy the front-end app to both Android and iOS smartphones. Also, Unity3D provides a spatial audio function using the HRTF method. PHP (version 8.3) and SQL were used for the back end, including user management functions and recording of daily duration of use. Figure 1 shows the main user interface of the Neglect Radio app. The source code for the front end (in Unity3D) and the back end (in SQL and PHP) is available on Zenodo under an open-source license [29].

To assess the usability of the app, 10 participants in the spatial hearing evaluation (see the Creating the Illusion of a Moving Sound Source section) were invited to test a prototype of the app on a smartphone. The testing procedure involved a 15-minute radio-listening session through the app, followed by completion of an online version of the System Usability Scale (SUS) [30], originally developed by Brooke [31] for usability testing.

### Ethical Considerations

This study was conducted in accordance with the Declaration of Helsinki. The research protocol and its amendments were reviewed and approved by the Kantonale Ethikkommission Bern (2020-02464; approval granted July 8, 2021) and the Ethikkommission Nordwest- und Zentralschweiz. The amendment “Effect of visuo-tactile enhanced audio-kinetic stimulation in virtual reality for neglect patients” was approved under an expedited review, with TN serving as coordinating investigator and sponsor.

Participants did not receive financial compensation or other incentives for participation. Written informed consent was obtained from all participants (or their legally authorized representatives, where applicable) prior to any study procedures. Participants were informed that participation was voluntary and that they could withdraw at any time. Data used for analysis were deidentified before access by the research team. Direct identifiers (eg, names, full dates of birth, and contact details) were removed and only the given scores for algorithms and the results of the SUS were kept.

## Results

### Spatial Realism Experiment

**Figure 2:**
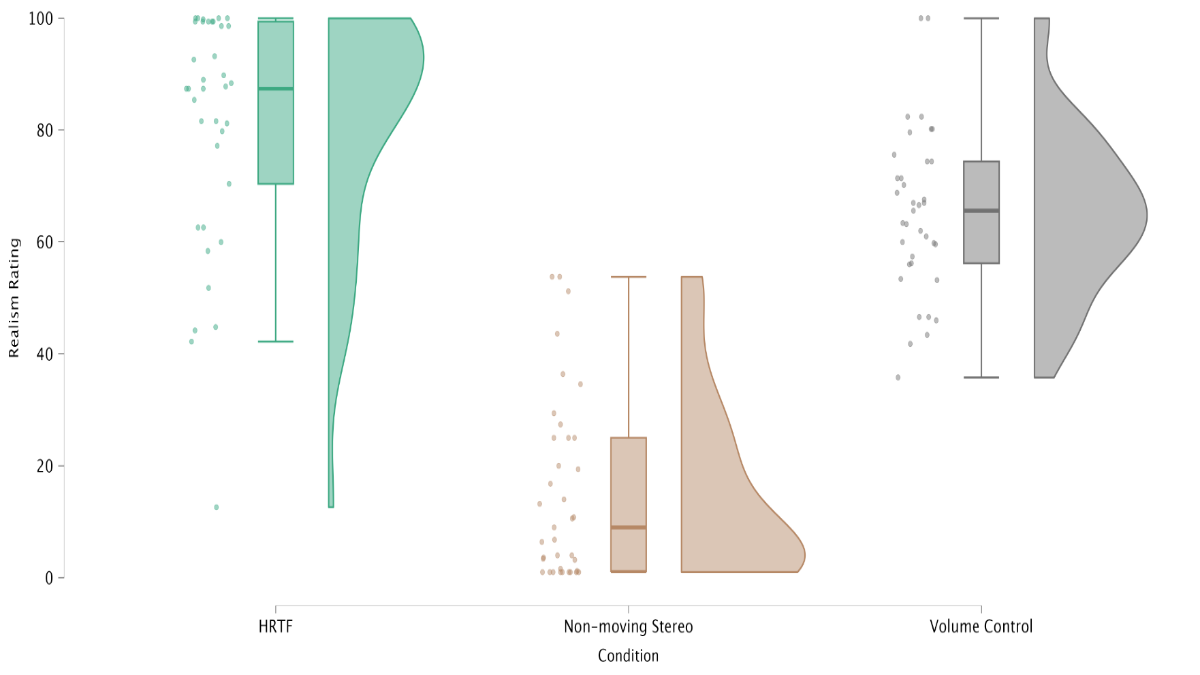
Realism ratings (0-100 scale) for 3 auditory conditions (nonmoving stereo, dynamic volume control, and head-related transfer function–based rendering) in an online perceptual study with 37 healthy volunteers recruited in Switzerland in 2025. Participants listened to 5 sound sources under each condition and rated the impression of sound movement (0=unrealistic, 100=highly realistic). HRTF: head-related transfer function.

A total of 37 volunteers participated in the online study, listening to 5 audio sources in 3 different conditions each (HRTF, nonmoving stereo, and volume control) and rating them with respect to the “impression that the sound source moves from the right to the left.” The evaluation was conducted on a scale from 0 to 100, with 0 indicating a very unrealistic and 100 a very realistic impression. Detailed descriptive statistics for each condition are presented in Table 1. Figure 2 illustrates the distribution of realism ratings across the 3 audio conditions.

**Table 1:** Descriptive statistics for realism ratings of 3 auditory motion conditions (static stereo, dynamic volume control, and head-related transfer function [HRTF]–based rendering) in 37 healthy participants recruited online in Switzerland in 2025. The study assessed perceptual realism of spatial auditory cues designed for potential application in hemispatial neglect rehabilitation after stroke.

	HRTF	Nonmoving stereo	Volume control
Valid responses, n	37	37	37
Missing responses, n	0	0	0
Realism rating, mean (SD)	80.9 (21.2)	15.2 (16.4)	65.1 (14.6)
Shapiro-Wilk test	0.833	0.823	0.976
*P* value for Shapiro-Wilk	< .001	< .001	.60
Realism rating, range	12.60-100.00	1.00-53.80	35.80-100.00

The realism rating for the HRTF-based rendering was significantly greater than for the volume control rendering (independent samples 1-tailed *t* test: t_72_=3.722; *P*<.001), and the rating for the nonmoving stereo condition was significantly lower than for the other two groups (*P*<.001). A repeated-measures ANOVA with Greenhouse-Geisser correction showed a significant main effect of condition on realism ratings (*F*_1.43,52.36_=120.6, *P*<.001; *F*_1.43, 52.36_=120.6, *P*<.001; *F*_1.43,52.36_=120.6, *P*<.001; Table 2). Mean realism ratings were highest for HRTF-based rendering (mean 80.9, SD 21.2), followed by dynamic volume control (mean 65.1, SD 14.6), and lowest for nonmoving stereo (mean 15.2, SD 16.4). Post hoc pairwise comparisons with Bonferroni correction confirmed that HRTF was rated significantly higher than volume control (*P*<.001), and both motion conditions (HRTF and volume control) were rated significantly higher than static stereo (both *P*<.001).

**Table 2:** Repeated-measures ANOVA of realism ratings across 3 auditory rendering conditions (static stereo, dynamic volume control, and head-related transfer function based). Reported are sums of squares (SS), dfs, mean squares (MSs), and F statistics for analyses without and with Greenhouse-Geisser correction. The Mauchly test indicated sphericity violation (*P*<.05); thus, Greenhouse-Geisser–corrected results are emphasized. dfs for F are shown as numerator and denominator; dfs in the MS column are those used to compute MS.

Cases and sphericity correction				SS (*df*)		MS		*F* test (*df*)		*P* value
**Within-subjects effects**										
	**Condition 1**									
		None	87,081^a^		43,540.4 (2.000)^a^		120.6 (1.43,52.36)^a^		< .001^a^	
		Greenhouse-Geisser	87,081		61,035.3 (1.427)		120.6 (1.43,52.36)		< .001	
	**Residuals**									
		None	25,990		361.0 (72.000)		—^b^		—	
		Greenhouse-Geisser	25,990		506.0 (51.362)		—		—	
**Between-subjects effects**										
	**Residuals**									
		—	7592		210.9 (36)		—		—	

^a^Mauchly test of sphericity indicates that the assumption of sphericity is violated (*P*<.05).

^b^Not applicable.

### Usability Evaluation

**Figure 3:**
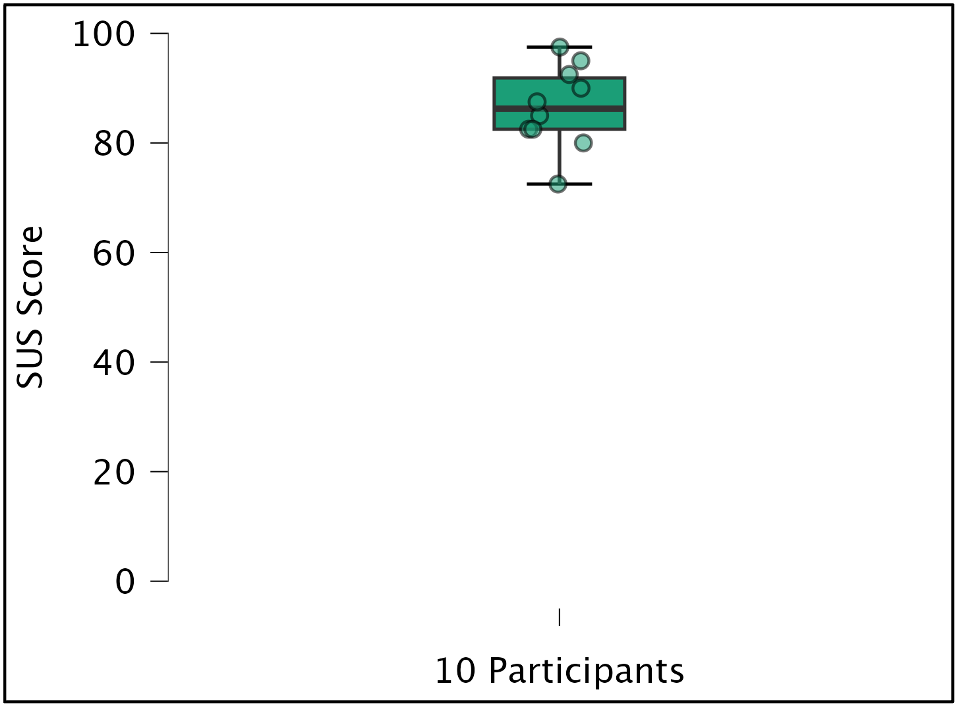
Individual System Usability Scale (SUS) scores.

Ten healthy volunteers tested the Neglect Radio smartphone app for 15 minutes in a laboratory-based preliminary evaluation focused on feasibility in healthy users prior to clinical testing with stroke patients with hemispatial neglect. The mean SUS score was 86.5 (SD 6.9), exceeding the 68-point threshold for above-average usability and a rating of “excellent.” [32]. The individual SUS scores are shown in Figure 3.

## Discussion

### Principal Findings

This study introduces Neglect Radio, a smartphone-based auditory motion stimulation app. We evaluated the realism of its sound and its usability with healthy participants. Realism ratings confirmed that HRTF-based rendering produced the strongest sense of spatial motion, outperforming simple volume control and static stereo. This finding suggests that leveraging HRTFs can create a compelling illusion of sound motion, likely because it reproduces the subtle timing and filtering cues that the brain expects from real-world sound sources [17,18]. In a rehabilitation context, higher perceptual realism may be important, since realistic spatial cues could engage patients’ attention more effectively and promote a stronger sense of motion [33]. The app also yielded an excellent mean SUS score of 86.5 (SD 6.9), corresponding to superior usability [34]. This indicates that users, even those unfamiliar with advanced technology, found the app intuitive, comfortable, and engaging to use. Such positive reception is encouraging, as it meets a relevant requirement for rehabilitation tools: if an intervention is not user-friendly, patients (especially older stroke survivors) are unlikely to adopt it in their daily routine [15]. Taken together, these results validate the core design choices of Neglect Radio: using HRTF-based spatial sound to maximize realism and delivering the intervention via a smartphone app that patients can easily operate.

Importantly, these results should be interpreted within the broader landscape of neglect rehabilitation. Traditional approaches have focused heavily on visual scanning training and visuospatial cueing as top-down methods, with mixed evidence for long-term benefits [35]. Although visual scanning remains a widely recommended strategy, reviews highlight its limitations when used alone and the need for complementary modalities [35,36]. Multisensory stimulation, particularly using a bottom-up approach (eg, prism adaptation, limb activation, or optokinetic stimulation), has been proposed to enhance treatment effects by engaging additional sensory channels [36,37].

In this context, Neglect Radio introduces auditory motion stimulation as a novel mobile health–based tool. Unlike purely visual methods, auditory cues do not require intact visual processing or continuous effortful scanning, which is often impaired by anosognosia or attentional deficits in patients with neglect [35,37]. By leveraging spatialized sound, auditory motion may reach patients who cannot reliably engage with visual-only rehabilitation tasks, thereby broadening accessibility. This aligns with evidence that multisensory stimulation can lead to stronger and longer-lasting improvements than unimodal training [36,37].

The possible clinical potential of the Neglect Radio app lies in its accessibility and scalability, providing auditory stimulation to patients irrespective of their location, even allowing home-based use. Its intuitive interface makes it suitable for older users, aligning with real-world usability needs. Integrating this technology into existing rehabilitation protocols could enhance therapeutic outcomes by offering continuous attention training, from inpatient to home-based settings. Furthermore, the passive logging of app use provides clinicians with valuable adherence data, enabling them to deliver personalized care.

Beyond the clinical rationale, delivering auditory motion stimulation through a smartphone offers clear scalability advantages compared with clinic-based visual or multimodal interventions. Other mobile apps for neglect, such as Neglect App and Negami, have primarily targeted visual exploration tasks [38,39]. Similarly, music- or rhythm-based rehabilitation apps (eg, GotRhythm) and cognitive telerehabilitation apps (Rehastart) have shown that smartphones can effectively deliver therapy and achieve high usability [40,41]. Against this backdrop, Neglect Radio contributes a unique auditory-focused approach, which may synergize with existing digital interventions to create richer, multimodal rehabilitation strategies.

### Limitations

The primary limitation is that no patients with stroke were included; findings are therefore restricted to feasibility in healthy volunteers. This limitation is partially addressed by the work by Geiser et al [14,15], who used an early prototype for clinical evaluation, confirming similar feasibility results in patients with stroke.

Also, exposure time was short (15 minutes) and the sample sizes were modest (n=37 for realism; n=10 for usability). While these are typical limitations in perceptual and usability research, they limit conclusions about long-term adherence or variability in patient populations. Finally, the online study could not fully standardize sound levels across devices, although relative levels were controlled.

In addition, the online testing environment prevented full control over listening devices, volume levels, and background noise, which may have influenced realism ratings. The modest sample sizes, while typical for feasibility work, limit generalizability, and long-term adherence could not be assessed. We did not collect qualitative feedback, which would have provided additional insight into user experience. Finally, the technical implementation is dependent on headphones, and different headphone models or device types may affect the accuracy of spatial rendering.

Future research should focus on clinical validation through controlled trials involving patients with neglect and assess long-term adherence, patient engagement, and functional outcomes. Additionally, refinement of content personalization, integration of multimodal stimuli (eg, augmented reality or vibrotactile cues), and enhanced app customization could further optimize patient outcomes. Longitudinal studies are also needed to assess sustained user engagement and therapeutic benefits.

### Conclusions

Neglect Radio provides a technically feasible and user-friendly platform for auditory motion stimulation. While healthy participants judged the stimuli as realistic and the app as highly usable, these results do not imply clinical efficacy. Future controlled clinical trials that include patients with stroke are essential to determine therapeutic effectiveness, adherence over time, and integration into rehabilitation practice.

## Appendix

Multimedia Appendix 1 CONSORT-eHEALTH checklist (V 1.6.1).

## Abbreviations

HRTF: head-related transfer function
SUS: System Usability Scale
